# The Importance of Physical Activity Exercise among Older People

**DOI:** 10.1155/2018/7856823

**Published:** 2018-12-05

**Authors:** Birgitta Langhammer, Astrid Bergland, Elisabeth Rydwik

**Affiliations:** ^1^Oslo Metropolitan University, Faculty of Health Sciences and Sunnaas Rehabilitation Hospital, Oslo, Norway; ^2^Oslo Metropolitan University, Faculty of Health Sciences, Oslo, Norway; ^3^Karolinska Institutet, Department of Neurobiology, Care Sciences and Society, Division of Physiotherapy, Huddinge, Sweden; ^4^Stockholm County Council, Research and Development Unit for the Elderly, Järfälla, Sweden

In this special issue of BioMed Research International, the focus is on lifestyle and in particular physical activity (PA) as a driver for a healthy and long life for older people.

As populations continue to extend life expectancy, a central concern is whether the added time comprises years of healthy life and promotes a high health-related quality of life into old age.* PA *is defined as any bodily movement produced by skeletal muscles that result in energy expenditure. PA encompasses exercise, sports, and physical activities performed as part of daily living, occupation, leisure, or active transportation.* Exercise* is a subcategory of PA that is planned, structured, and repetitive and that has as a final or intermediate objective for improvement or maintenance of physical fitness.* Physical function* is the capacity of an individual to perform the physical activities of daily living. Physical function reflects motor function and control, physical fitness, and habitual PA [1].

PA is a protective factor for noncommunicable diseases such as cardiovascular disease, stroke, diabetes, and some types of cancer [2] and PA is associated with improved mental health [3], delay in the onset of dementia [4], and improved quality of life and wellbeing [5, 6]. The health benefits of PA are well documented with higher levels and greater frequency of PA being associated with reduced risk and improved health in a number of key areas [7].

The dose of PA or exercise is described by the duration, frequency, intensity, and mode [8]. For optimal effects, the older person must adhere to the prescribed exercise program and follow the overload principle of training, i.e., to exercise near the limit of the maximum capacity to challenge the body systems sufficiently, to induce improvements in physiological parameters such as VO2max and muscular strength [1].

Improvements in mental health, emotional, psychological, and social well-being and cognitive function are also associated with regular PA. Despite these health benefits, PA levels amongst older adults remain below the recommended 150 min/week [9]. The crude global prevalence of physical inactivity is 21.4% [10]. This translates to one in every four to five adults being physically inactive, or with activity levels lower than the current recommendations from WHO [11]. Inactivity and aging increase the risk of chronic disease, and older people often have multiple chronic conditions (NFH, 2010). The exercise recommendations from WHO include both aerobic exercise and strength exercise as well as balance exercises to reduce the risk of falls. If older adults cannot follow the guidelines because of chronic conditions, they should be as active as their ability and conditions allow [12]. It is important to note that the recommended amount of PA is in addition to routine activities of daily living like self-care, cooking, and shopping, to mention a few.

Inactivity is associated with alterations in body composition resulting in an increase in percentage of body fat and a concomitant decline in lean body mass. Thus, significant loss in maximal force production takes place with inactivity. Skeletal muscle atrophy is often considered a hallmark of aging and physical inactivity. Sarcopenia is defined as low muscle mass in combination with low muscle strength and/or low physical performance [13]. Consequently, low physical performance and dependence in activities of daily living is more common among older people [14, 15]. However, strength training has been shown to increase lean body mass [16], improve physical performance [17, 18], and to a lesser extent have a positive effect on self-reported activities of daily living [18]. These aspects are at focus in the papers of K. Kropielnicka et al. “Influence of the Physical Training on Muscle Function and Walking Distance in Symptomatic Peripheral Arterial Disease in Elderly” as well as G. Piastra et al. “Effects of Two Types of 9-Month Adapted Physical Activity Program on Muscle Mass, Muscle Strength, and Balance in Moderate Sarcopenic Older Women.”

Participation in PA and exercise can contribute to maintaining quality of life, health, and physical function and reducing falls [19–21] among older people in general and older people with morbidities in particular. The increased attention to the relationship between exercise and HRQOL in older adults over the last decade is reflected in a recent review, which showed that a moderate PA level combining multitasking exercise components had a positive effect on activities in daily living, highlighting the importance of physical, mental, and social demands [22]. To reduce falls, balance training is also recommended to be included in physical exercise programs for older adults [12]. Exercise has also been shown to reduce falls with 21%, with a greater effect of exercise programs including challenging balance activities for more than 3 hours/week [23].

The gender perspective and motivators for fall prevention are at focus in M. Sandlund et al. qualitative study “Gender Perspective on Older People's Exercise Preferences and Motivators in the Context of Falls Prevention: A Qualitative Study,” in this special issue.

Exercise training in older people has been associated with health benefits such as decreased cardiovascular mortality [24]. Explanatory mechanism likely to be involved following exercise was a change in the cardiac autonomic balance producing an increase, or a relative dominance, of the vagal component [25]. Furthermore, endurance exercise training in older people decreases resting and submaximal exercise heart rate and systolic and diastolic blood pressure and increases stroke volume [26]. This is especially notable during peak effort in which stroke volume, cardiac output, contractility, and oxygen uptake are increased, while total peripheral resistance and systolic and diastolic blood pressure decreased. Thus lowering after-load in the heart muscle, which in turn facilitates left ventricular systolic and diastolic function, emphasizes the importance of high intensity training also for the elderly. E. Tamuleviciute-Prasciene et al. focus on the frail elderly individuals and exercise in their contribution “Frailty and Exercise Training: How to Provide Best Care after Cardiac Surgery or Intervention for Elder Patients with Valvular Heart Disease.”

Exercise may also have benefits for the brain centers that support executive control. It may be that strong executive functioning in itself may facilitate consistency for this challenging activity. Poor executive control has been associated with lower self-reported PA rates over a 2-year period [27, 28]. The executive control's contribution to PA has been found to be 50% greater in magnitude than the contribution of PA to subsequent changes in executive control [29]. In the paper of M. A. McCaskey et al. “Making More of IT: Enabling Intensive MOtor Cognitive Rehabilitation Exercises in Geriatrics Using INFORMATION Technology Solutions,” the authors also include new technology to enhance and maintain exercise in cognitive rehabilitation.

In order to attain a high level of cardiorespiratory fitness, it is recommended to be physically active for 6 months or longer. These recommendations may also be applied to balance exercises in order to reduce falls [23]. Many elderly individuals are incapable of sustaining activities for this long on their own. Successful maintenance of PA typically requires substantial support and supervision. Even then, a high percentage of people drop out due to difficulties negotiating everyday costs of activity participation like scheduling conflicts and competing sedentary activities or health issues. This issue is highlighted in the study of T. Adachi et al. “*Predicting the Future Need of Walking Device or Assistance by Moderate to Vigorous Physical Activity: A 2-Year Prospective Study of Women Aged 75 Years and Above.”*

In addition, reduced bodily functions can make it difficult for elderly persons to maintain exercise under different environmental circumstances, which is demonstrated in the contribution of B. N. Balmain et al. “*Aging and Thermoregulatory Control: The Clinical Implications of Exercising under Heat Stress in Older Individuals*.”

In this special issue, we have included papers that focus on the aging process and PA in a broad perspective, focusing on different aspects on PA, exercise, and older people. PA and exercise play an important role in the primary, secondary, and tertiary prevention, in the management of diseases, to counteract sarcopenia and falls as well as improving physical performance and activities of daily living, as these papers illustrate.

Promoting exercise among the older population is an important public health and clinical issue. A core issue is how to get older people with comorbidities to exercise.
